# Impact of a community-based intervention on *Aedes aegypti* and its spatial distribution in Ouagadougou, Burkina Faso

**DOI:** 10.1186/s40249-020-00675-6

**Published:** 2020-06-05

**Authors:** Emmanuel Bonnet, Florence Fournet, Tarik Benmarhnia, Samiratou Ouedraogo, Roch Dabiré, Valéry Ridde

**Affiliations:** 1 Résiliences, French National Research Institute for Sustainable Development, 32 Avenue Henri Varagnat, 93140 Bondy, France; 2 Infectious Diseases and Vectors Ecology, Genetics, Evolution and Control (MIVEGEC), French National Research Institute for Sustainable Development, 911 Avenue Agropolis, BP 64501, 34394 Montpellier Cedex 5, France; 3 University of California, San Diego, CA USA; 4 University of Montreal Public Health Research Institute, Montreal, Canada; 5 Institute for Health Science Research, Bobo-Dioulasso, Burkina Faso; 6 Population and Development Center (CEPED), French National, Research Institute for Sustainable Development, Université Paris Sorbonne, 45, rue des Saints Pères, 75006 Paris, France

**Keywords:** Vector-borne diseases, Spatial analysis, Community-based intervention

## Abstract

**Background:**

Several studies highlighted the impact of community-based interventions whose purpose was to reduce the vectors’ breeding sites. These strategies are particularly interesting in low-and-middle-income countries which may find it difficult to sustainably assume the cost of insecticide-based interventions. In this case study we determine the spatial distribution of a community-based intervention for dengue vector control using different entomological indices. The objective was to evaluate locally where the intervention was most effective, using spatial analysis methods that are too often neglected in impact assessments.

**Methods:**

Two neighbourhoods, Tampouy and Juvenat in Ouagadougou, Burkina Faso, were chosen among five after a survey was conducted, as part of an assessment related to the burden of dengue. As part of the community-based intervention conducted in Tampouy between August and early October 2016, an entomological survey was implemented in two phases. The first phase consisted of a baseline entomological characterization of potential breeding sites in the neighbourhood of Tampouy as well as in Juvenat, the control area. This phase was conducted in October 2015 at the end of the rainy season. The mosquito breeding sites were screened in randomly selected houses: 206 in Tampouy and 203 in Juvenat. A second phase took place after the intervention, in October 2016. The mosquito breeding sites were investigated in the same yards as during the baseline phase. We performed several entomological analyses to measure site productivity as well as before and after analysis using multilevel linear regression. We used Local Indicators of Spatial Association (LISAs) to analyse spatial concentrations of larvae.

**Results:**

After the intervention, it is noted that LISAs at Tampouy reveal few aggregates of all types and the suppression of those existing before the intervention. The analysis therefore reveals that the intervention made it possible to reduce the number of concentration areas of high and low values of pupae.

**Conclusions:**

The contribution of spatial methods for assessing community-based intervention are relevant for monitoring at local levels as a complement to epidemiological analyses conducted within neighbourhoods. They are useful, therefore, not only for assessment but also for establishing interventions. This study shows that spatial analyses also have their place in population health intervention research.

## Background

Whilst many countries in both Africa and Asia are well on the way to eliminating malaria, the world has faced frequent outbreaks of arboviral diseases since 2015, including the recent epidemics of yellow fever in Brazil (2017), the Democratic Republic of Congo (2016) and Angola (2015), or of Zika in Latin America [1–3]. This increase in outbreaks of arboviral diseases, especially of chikungunya and dengue [4], which impact high-income countries (HIC), is driven mainly by global changes, demographic expansion, trade exchanges, international mobility of populations and urbanization. These factors favour the spread of the viruses and their mosquito vectors, mainly *Aedes aegypti*, a species particularly well adapted to human environments.

In this regard, the use of spatial methods in epidemiology and public health remains low in countries of the Global South. One of the reasons is linked to the underuse of geo-tracking of epidemiological and entomological data [5]. A recent literature review shows that since 2008, recourse to spatial methods has increased and expresses the need for more in-depth knowledge in the face of an increase in epidemics in countries of the Global South [6]. Spatial analyses effectively make it possible to gain a better understanding of the dynamics of transmission and therefore to formulate strategies to combat vectors. The purpose of this paper is to mobilize spatial methods to enable evaluation of the effects of community action to combat dengue in Burkina Faso.

In Burkina Faso, selvatic circulation of the dengue virus was reported a long time ago [7]. Urban circulation was reported in 1986 [8] around Bobo-Dioulasso, the second largest city in the country, and an outbreak occurred in the capital city, Ouagadougou, in 1987 [9]. When a dengue outbreak occurred in Ouagadougou in 2013, it was poorly received [10]. The identification of the virus showed that serotype 2 was involved, as it was in the 1980s, but at least two other serotypes, 3 and 4, were also identified [11, 12].

To date, only yellow fever can be effectively controlled by vaccination. Recently, a vaccine against the dengue virus was registered by several national regulatory agencies; nevertheless, the performance and security of this vaccine are under discussion and the vaccine is not routinely implemented [13]. Consequently, the main strategy to control and prevent dengue and other arboviral diseases remains vector control and avoiding bites from mosquitoes. This can be achieved through different means such as the use of larvicides to control immature mosquitoes, insecticide-treated materials to prevent the entry of adult mosquitoes into houses and also elimination of breeding sites [14]. Such interventions require knowledge of existing vectors, particularly in terms of abundance. However, apart from a rapid entomological investigation in 2004 following a yellow fever outbreak in Bobo-Dioulasso [15], there have been no entomological studies conducted in Ouagadougou [16, 17].

Several studies highlighted the impact of community-based interventions (CBIs) [18] whose purpose was to reduce the vectors’ breeding sites [19, 20]. These strategies are particularly interesting in low-and-middle-income countries which may find it difficult to assume the cost of insecticide-based interventions sustainably.

After the first outbreak of dengue in Ouagadougou in 2013, an intervention was planned and implemented in a neighbourhood of the city in 2016. This intervention was based on community mobilization to reduce the larval source for *Ae. aegypti* and included educational campaigns to protect people from dengue [21]. We evaluated the effectiveness of this community-based intervention for combatting vectors and changing attitudes, providing knowledge and competences connected with dengue on the part of indigenous populations in Ouagadougou. This intervention was developed together with local contributors and adapted for the community using the EcoHealth approach. The contents took into account the most recent evidence-based findings on which actions to implement in order to reduce the number of vectors responsible for dengue. Analyses showed that the intervention had reduced the area’s residents’ exposure to *Ae. aegypti* mosquito bites as well as Breteau indices in particular. These findings also showed that the communities’ knowledge of measures to combat mosquitoes had improved in the intervention zone but not in the control zone.

In this new article, which complements the evaluation of the CBI, we aimed to determine the spatial distribution of vector control impacts using different entomological indices. The objective was to evaluate locally where the intervention was most effective, using spatial analysis methods that are too often neglected in impact assessments.

## Materials and methods

### Study site

Ouagadougou (12°21′14″ N, 1°30′41″ W) is the capital of Burkina Faso in West Africa. In 2006, the national census reported a population of around 1 million inhabitants [22]. There has not been a new census of the population since 2006, but according to National Institute of Statistics and Demography and World Bank estimates [23], Ouagadougou is estimated to have over 2 million inhabitants. Since 2007, urbanization has accelerated with a significant regularization of the periphery, but neighbourhoods still encounter serious difficulties with respect to access to urban services, with the capital experiencing frequent power and water cuts. The annual average rainfall is between 600 and 900 mm, the rainy season occurring from May to October.

The intervention [21, 24] was derived from selected CBIs that have proven effective in the control of dengue, and through a participative process with community leaders. The intervention neighborhood received a behavior change intervention. Community leaders invited the population to participate in the intervention. Interested persons attended communication and education activities, including a community theater. The intervention also included door-to-door visits, school education, and self-awareness assessment sessions. In the control area, no activities were organized out for dengue awareness and control.

Two neighbourhoods, Tampouy and Juvenat, about 12 km apart, were chosen among five after a survey was conducted, as part of an assessment related to the burden of dengue, with the support of the Dengue Vaccine Initiative. Tampouy was randomly selected to receive the intervention and Juvenat to be a control area. Tampouy is located in the north-west part of the city and Juvenat on the eastern side. They shared similar socio-economic characteristics. There are affluent households living in modern houses with running water and electricity alongside households enjoying a modest standard of living, and poor people living in modest clay houses, often lacking such basic commodities. The principal issue in this area is one of sanitation due to poor waste management and street cleaning. Many residents dispose of their rubbish and sewage in the street, which encourages mosquito breeding sites.

### Study design

As part of the community-based intervention conducted in Tampouy between August and early October 2016, an entomological survey was implemented in two phases. The first phase consisted of a baseline entomological characterization of potential breeding sites in the neighbourhood of Tampouy as well as in Juvenat, the control area. This phase was conducted in October 2015 at the end of the rainy season. The mosquito breeding sites were screened in randomly selected houses: 206 in Tampouy and 203 in Juvenat. A second phase took place after the intervention, in October 2016, also at the end of the rainy season. The mosquito breeding sites were investigated in the same yards as during the baseline phase.

### Data collection

#### Entomological data

All domestic recipients and containers containing water were recorded and classified according to four categories as: discarded containers, water storage containers, used tyres, and various other recipients. The presence or absence of any immature mosquito from each container was reported and the larvae and pupae were collected and stored in jars identified with the yard reference number. A survey sheet was filled in with the yard identifier, the number of residents in the yard, the different breeding sites with their type and status (with or without mosquito aquatic stages). Back in the laboratory, mosquito aquatic stages were counted and dispatched between species (*Culex, Aedes* or *Anopheles*). *Aedes* stages were distinguished: larvae or pupae. All immature *Aedes* were transferred to larva breeding containers in the laboratory to continue their development until adulthood. After emergence, adults were counted and the species identified on the basis of morphological criteria using the identification keys of [25, 26]. A database was created with all the collected variables including the geographical coordinates of the yard.

The different types of breeding site were analysed to evaluate a potential modification of population behaviours. The productivity of each kind of breeding site was not evaluated. *Stegomyia* indices i.e. container index (CI, percentage of recipients positive for larvae and/or pupae), house index (HI, percentage of houses positive for larvae and/or pupae), Breteau Index (BI, number of positive containers per 100 houses), and pupae per person index, were computed at the baseline and after the intervention at endline.

### Statistical analysis

We analysed the change in the overall proportion of positive containers for *Aedes* aquatic stages per household before and after the intervention. We quantified a within-household change (before and after) in the proportion of positive containers for mosquito aquatic stages as an aggregated measure of these entomological indices used in this study (see details above). We used a multilevel linear regression with a fixed effect at the neighbourhood level Analyses were conducted by using Stata 14.2 (StataCorp LLC, College Station, TX, USA).

### Spatial analysis

In order to evaluate the effects of the intervention on the control of vectors, descriptive and statistical spatial analyses were performed on the sum of the number of larvae per inhabitant at the concession level which also represented the number of positive breeding sites. This variable was retained as it reveals the productivity relating to a spatially comparable unit, the dwelling place.

- Mapping the sum of the number of larvae per inhabitant of the concession
- Calculation of the global coefficient of Moran’s spatial autocorrelation which indicates if the distribution of the CI index is clustered, dispersed or random in space
- Local Indicators of Spatial Association (LISA) used for a spatial exploration enabling the detection of a localized spatial structure, i.e. a high or low spatial concentration of the values of the CI index.

The descriptive spatial analysis of the number of larvae per inhabitant, at the household level, was produced by mapping the values at the location of households in each of the neighbourhoods, before and after the intervention. The choice of graphical representation is defined by the practices outlined in numerous works dedicated to graphic semiology [27] and developed with the ArcGis 10.5 (Esri, Inc., Redlands, CA, USA).

To examine spatial and temporal trends, both local and global clustering techniques were used [28, 29] such as spatial autocorrelation which is a global measurement making it possible to determine whether there is a correlation between the value of objects and the metric or topographical relationships between these objects. To account for the neighbouring values, one uses indices of correlation, such as the Moran index. It is defined by the average of the products of normalized values of pairs of points, weighted by the distance between two points [29].

$$ I=\frac{N\sum \limits_{i=1}^n\sum \limits_{j=1}^n{W}_{ij}\ \left({x}_i-\overline{x}\right)\left({x}_j-\overline{x}\right)}{\left(\sum \limits_{i=1}^n\sum \limits_{j=1}^n{W}_{ij}\right)\ \sum \limits_{i=1}^n{\left({x}_i-\overline{x}\right)}^2} $$

*Where:*

*N is the number of observation (points or polygons)*

$\overline{x}$*is the mean of the variable*

*x*_*i*_*is the variable value at a partiular location*

*x*_*j*_*is the variable value at another location*

*W*_*ij*_*is a weight indexing location of i relative to j*

**Formula for Moran’s *I*(Briggs Henan University 2010)**

However, Moran’s index can hide the spatial heterogeneity on local scales, insofar as the index is an average of the spatial model over the whole of the study area. The value of spatial autocorrelation is therefore an initial true indicator which should be examined in depth at the local level.

Local indicators of spatial association developed by Luc Anselin [29] allow for these evaluations. They make it possible to analyse the concentration of similar and dissimilar values measured on a whole set of points or calculated by spatial aggregation.

$$ {I}_i=\frac{\sum \limits_j{w}_{ij}\left({p}_i-\overline{p}\right)\left({p}_j-\overline{p}\right)}{\sum \limits_i{\left({p}_i-\overline{p}\right)}^2} $$

- *p*_*i*_*and p*_*j*_*the values of spatial units i and j such that i and j are considered as neighbours* given the measurement of their degree of proximity
- *p*_*i*_*the mean value of spatial units*
- *w*_*ij*_*a measurment of the proximity of spatial units i and j*

The analyses were done using the free and open source software GeoDa. A spatial weights matrix (a chart which identifies all the distances between all neighbours) was used with a threshold distance of 300 m so that the analysis should take into account at least one neighbour [29]. The interpretation of LISAs obtained is based on statistical assumptions of normality. It results in five cases: the first is when value p provides no basis to reject the null hypothesis, the point is not considered as significant to be aggregated with another point, and four other cases whose LISA indices are used for a typology of four aggregates according to the value of the individual and the value of the neighbourhood. These aggregates are represented below (Table 1), with the colour generally used in their mapping.

**Table 1:**
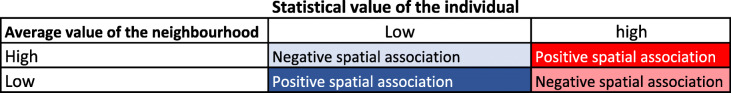
The four types of spatial association aggregations [29]

## Results

### Entomological findings

The number of immature instars (larvae and pupae) of *Ae. aegypti* stood at 9096 before the intervention (4419 in Juvenat and 4677 in Tampouy), and at 6063 after the intervention (3940 in Juvenat, 2123 in Tampouy). There were significantly fewer immature stages in Tampouy after the intervention (*t* = 2.362; *P* = 0.0186) compared to Juvenat .

In water-holding containers, immature instars of *Ae. aegypti* varied according to the type of recipient (Table 2). Before the intervention, *Ae. aegypti* were mainly collected in discarded recipients (plastic pots, used tyres, tins) in the control area (68.8% of positivity) as well as in the intervention area (62.4% of positivity). Water storage containers were also frequently used as breeding sites by the mosquitoes in both areas (27.5 and 36.5% respectively in control and intervention areas). The intervention seemed to have an impact on the *Ae. aegypti* larval population in the water storage recipients in the intervention area (69.4% of reduction), but not on the discarded containers (5.7% increase).

**Table 2:** Distribution of positive recipients according to the neighbourhoods and the intervention

	Control			Intervention		
Recipient types (%)	Baseline	Endline	***Difference***	Baseline	Endline	***Difference***
**Water storage**	27.5 (30)	27.7 (28)	*+ 6.7*	36.5 (31)	12.3 (7)	*-69.4*
**Discarded containers**	68.8 (75)	72.3 (73)	*+ 2.7*	62.4 (53)	87.7 (50)	*+ 5.7*
**Miscellaneous**^**a**^	3.7 (4)	0 (0.0)	*-100.0*	1.2 (1)	0 (0.0)	*-100.0*
	109	98		85	57	

^a^ water flows and puddles

In Tampouy, the entomological indices (HI, CI, BI and pupae per person) were lower after the intervention (Table 3).

**Table 3:** Comparison of entomological indices at baseline and endline according to the districts

	No. positive houses	No. positive containers	No. larvae	No. pupae	HI	CI	BI	Pupae per person index
**Baseline**								
Control	67	109	3975	444	33.0	28.8	54.2	0.225
Intervention	66	85	4343	334	32.0	12.3	41.9	0.191
**Endline**								
Control	65	98	3421	519	32.0	36.3	48.3	0.260
Intervention	44	57	1919	204	21.4	10.8	27.7	0.133

*CI* Container index, *HI* house index, *BI* Breteau Index

### Statistical analysis

We quantified a within-household change (before and after) in the proportion of containers found to be positive for pupae. We included 242 households in our analysis. The average difference between the intervention and the control zones was 9.67% (95% confidence interval [*CI*]: 1.1–18.3%) of change in the proportion of recipients found to be positive for larvae by households indicating a potential impact of the intervention.

### Spatial analysis

#### Mapping

**Fig. 1:**
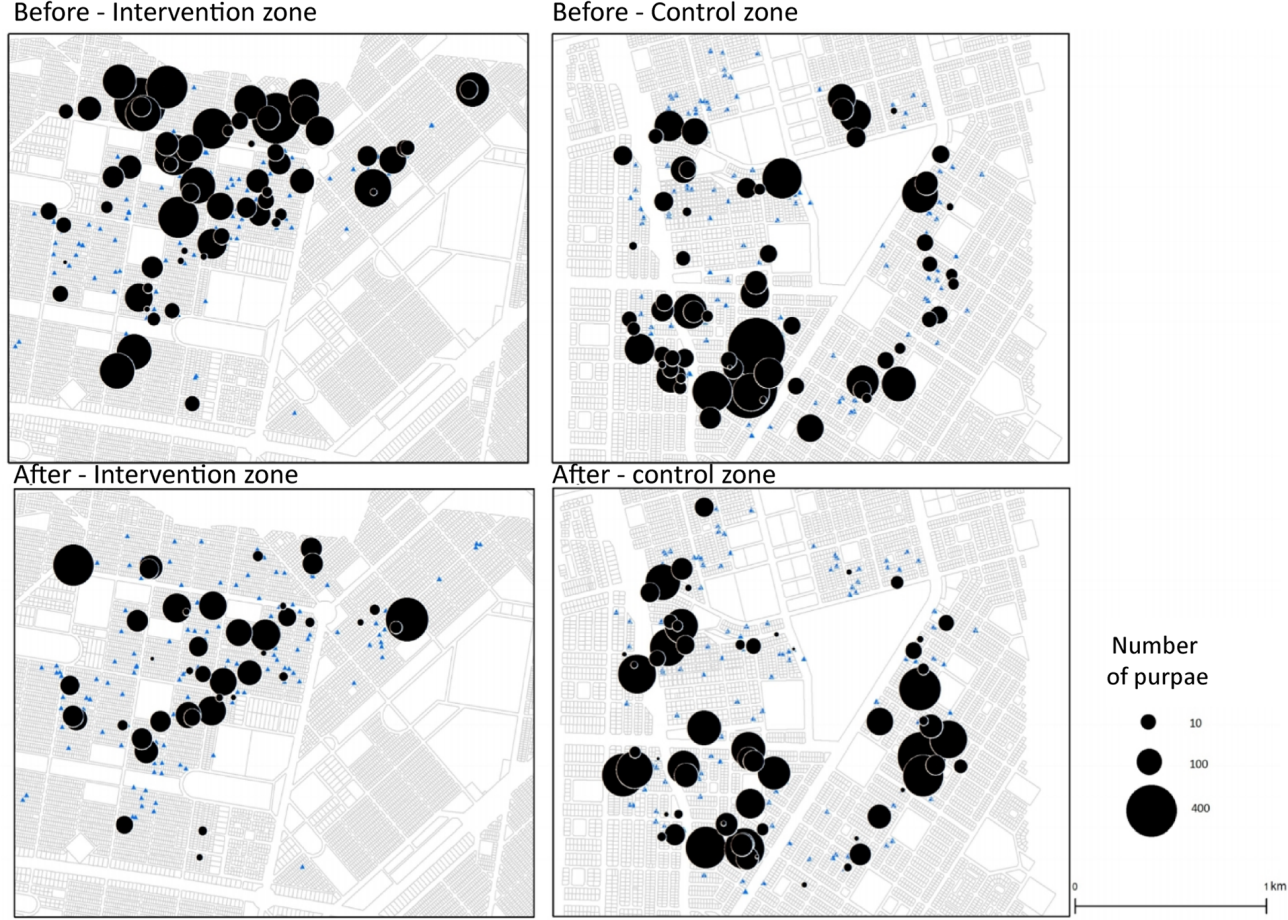
Mapping of the number of pupae per household in the study areas pre- and post-intervention

Maps representing all the households (*n* = 206 in Tampouy and *n* = 203 in Juvenat) where the data were collected. If no larvae were collected, the household is represented by a blue dot on the map, expressing no quantity (Fig. 1). As regards the other households, the black dot is graphically proportional to the number of pupae.

During the pre-intervention stage, a large number of pupae can be observed in the intervention zone (Tampouy) and the control zone (Juvenat). After-intervention analyses show a substantial diminution in the number of households with positive breeding sites in the intervention zone. We can visualize fewer circles and a smaller size of these circles. This shows a decrease in the number of households with positive gites, but also a decrease in the number of breeding sites in the households still exposed. In the control zone, it can be seen that the breeding sites identified during the initial phase are mostly present, as well as the appearance of new breeding sites. It should be noted (Appendix) that the effects are not associated with a lower rainfall in one zone as compared to another, and from 1 year to the next, and therefore to a lower productivity of larval breeding sites.

#### Global spatial autocorrelation

The Moran value index on the baseline number of pupae in the intervention zone shows a positive spatial autocorrelation (i = 0.103), but with a dispersion of index values which suggests that possible aggregates are present. After the intervention, it can be observed that the spatial autocorrelation is null (i = -0.02) which signifies that the values are distributed in random fashion, without autocorrelation, and reveals an evolution between the two collection phases and therefore an intervention effect. In the control sector, the global spatial autocorrelation is also positive (i = 0.05) before the intervention and null (i = -0.004) afterwards. The global evolution in the two zones is thus similar. However, it is important that the spatial autocorrelation at a global level be verified at local level, i.e. at the householders’ houses, to determine if aggregates exist, and if they do, how they have evolved.

#### LISA

**Fig. 2:**
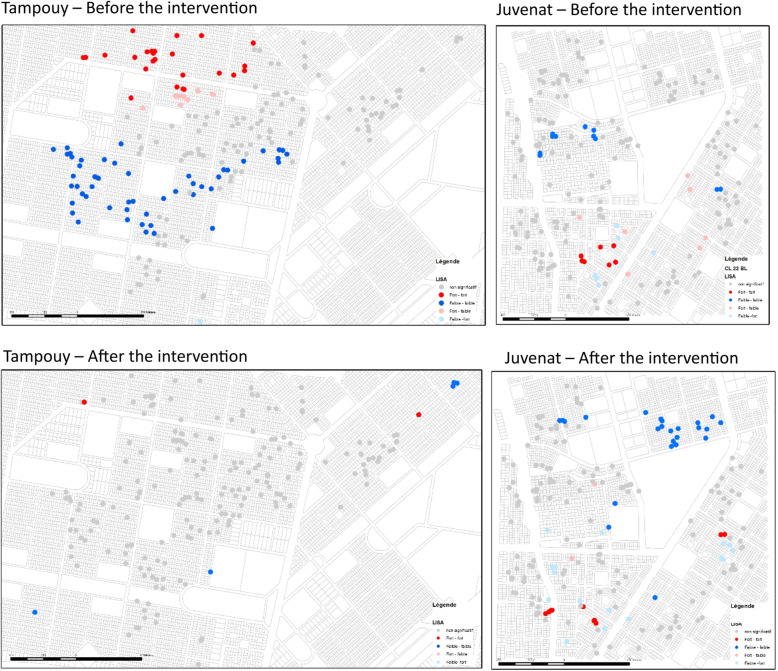
LISA Endline: Suppression of existing aggregates

The LISA analyses (Fig. 2) on the number of pupae, done for the intervention zone (on the left), show that before the intervention there were negative value aggregates (in blue) and positive values (in red) present. There were therefore two very distinct types of concentrations in the neighbourhood. In blue, a concentration of households with breeding sites containing pupae, albeit relatively unproductive (low number of pupae). This concentration of low-low values means also that neighbouring households had similar characteristics (low number of larvae). Conversely, north of the zone, the concentration of high values is represented by points classified as high-high (high values of the household and its neighbours). LISA therefore reveals a twofold concentration of opposing values of larvae. This does not mean a presence or absence of pupae, but graduated zones of pupae production in the area studied before the intervention.

After the intervention, it is noted that LISAs at Tampouy reveal few aggregates of all types and the suppression of existing aggregates before the intervention. The analysis therefore reveals that the intervention made it possible to reduce the number of concentration areas of high and low values of pupae. The global analysis confirms this with a null spatial autocorrelation, which means that from now on there is a random presence of larvae in the intervention zone. This analysis completes the epidemiological analyses which have shown a low reduction in indices in the intervention zone after its implementation (Table 2). In the control area, aggregates can be seen to persist in the same places or nearby.

LISA: Local Indicators of Spatial Association.

## Discussion

There is very little academic literature that deals with the spatial heterogeneity of dengue and the way in which the vectors are distributed in inter-urban space and this, whatever the scale of the analysis [29]. It is important to note that the mapping and spatial analyses of this heterogeneity make it possible not only to plan effective control strategies and elimination programs, but also to assess their impact. The CBI evaluation carried out in Ouagadougou in 2016 [21] is supplemented by an innovative spatial analysis, in the sense that no evaluation of a community intervention has been carried out using these methods in Africa, and that it confirms the positive effect of the intervention while showing where it was most effective.

This approach is all the more interesting as the classic use of stegomian indices to assess the risk of dengue is increasingly discussed [30]. Effectively, the link between the values of the different indices and transmission of dengue is far from clear. And even if thresholds have been defined for evaluating the risk of dengue epidemization in a given site [31, 32], it may be necessary to question the importance of the values chosen in relation to an acceptable disease rate and in relation to the scale at which the thresholds should apply.

What is more, recent studies highlight the difficulty in appreciating the effectiveness of the measures deployed during the interventions [30, 33]. According to the studies, the same measures (waste management, eradication of breeding grounds) can have various effects. These difficulties illustrate the need to dispose of new tools to assess the situation which, of necessity, is known to be heterogeneous given the environment but also the practices of indigenous populations. Indeed, the suppression of spatial clusters of larval breeding sites can be interpreted as the proper application of measures to combat the vector that communities have implemented during community intervention, notably with respect to water storage containers. We did not find any major effects of the intervention upon the number of breeding sites or the number of larvae and pupae over the neighbourhood as a whole. However, it can be noted that if the number of breeding sites is still high there are no longer any court which concentrate a large number of breeding sites in their yards, and in the neighbours’ yards where there no data gathering has taken place. The intervention therefore did not appear to have an effect on the quantity of breeding sites and larvae, but on their concentration in the concessions. The application of measures to combat infestation thus appears better after the intervention but there is still room for improvement in order to eliminate larval breeding grounds more effectively. The intervention based on the most recent solid evidence and people’s preferences seems therefore to be effective in the neighbourhood as a whole in terms of improvement in residents’ knowledge, and effective within the concessions since the elimination of local clusters has been observed.

### Contribution of spatial methods for assessing community-based intervention

Among the existing set of spatial analysis methods, the LISAs and Moran’s Global Statistic remain the most widely used given their widespread availability in commercial software packages like the ubiquitous©ArcGIs or free ones like© GeoDa. Whatever the software and methods used, spatial analysis enhances analysis by producing effective information for detecting hotspots of dengue cases or larval breeding sites and therefore of potential transmission of arboviruses. These methods are therefore particularly relevant for monitoring at local levels as a complement to epidemiological analyses conducted within neighbourhoods. Finally, spatial analysis and associated mapping provide other opportunities for preventative programmes because by identifying the precise areas where vectors are concentrated, programmes for combating the vectors can be targeted by treating high-risk zones as a priority. They are useful, therefore, not only for assessment but also for establishing interventions.

The main limitation of the method is that it does not allow several variables to be mobilized at the same time, thus limiting the taking into account of other variables which could be considered as confounding factors. However, the variable used here is representative of the effects of the intervention on the combat against vectors. Another limitation concerns the duration of the analyses. They were conducted over relatively short durations (2 years) and preclude longer term analyses.

## Conclusions

The contribution of spatial methods for assessing community-based intervention are relevant for monitoring at local levels as a complement to epidemiological analyses. The assessment of the spatial dimension in public health interventions is often limited to an analysis of the average effects of an intervention which vary according to geographical entities and approximate measurements of distance. The evaluation methods traditionally employed do not make it possible to account for the spatial variability of the places and effects of interventions. Heterogeneity is not merely noise in the analyses as some economists like to say but is often a source of learning as this assessment of the intervention in Burkina Faso makes clear. These methods are rarely employed to evaluate interventions, more so to explore the spread or the concentration of a disease in space. Yet this example shows that the phenomena can be observed at different scales and the overall results can hide different local situations.

Evidence based community mobilization against *Aedes* vectors should be considered in integrated strategies combining new vaccines and innovative vector control technologies such as the use of transgenic mosquitoes, release of strains infected with *Wolbachia*, auto-dissemination of juvenile hormone artic mimics as well as the renewal of the Sterile Insect Technique, although all are some way from mass application [30]. However, to measure their effectiveness in the territories, interdisciplinary reflection is needed with researchers in statistics, public health, global health and parasitology allowing a broader analysis of the processes studied. Integration of geographical methods has become an important factor in public health and epidemiology today [34], but the example of this paper also shows that spatial analyses have their place in the processes of intervention assessment. However, these strategies of analysis should be based on monitoring systems, which are still rare [35], in particular in low-income countries.

## Supplementary information

**Additional file 1.**

**Additional file 2.** Questionnaire Endline.

## Data Availability

The datasets used and/or analysed during the current study are available from the corresponding author on reasonable request.

## Abbreviations

LISA: Local Indicators of Spatial Association
CI: Container index
HI: House index
BI: Breteau Index
*CI*: Confidence interval
