# Supplementary material for: Impact of a community-based intervention on Aedes aegypti and its spatial distribution in Ouagadougou, Burkina Faso
Source: Infect Dis Poverty. 2020 Jun 5;9:61. doi: 10.1186/s40249-020-00675-6 (PMC7275586; doi:10.1186/s40249-020-00675-6)

Appendix

In Ouagadougou, the annual average rainfall range from 600 to 900 mm, from May to October. Les deux années d'études sont similaires en quantité totale de pluie et restent dans les normes connues pour Ouagadougou. On note un début précoce des pluies et une réduction de la saison des pluies en 2016 par rapport à 2015.

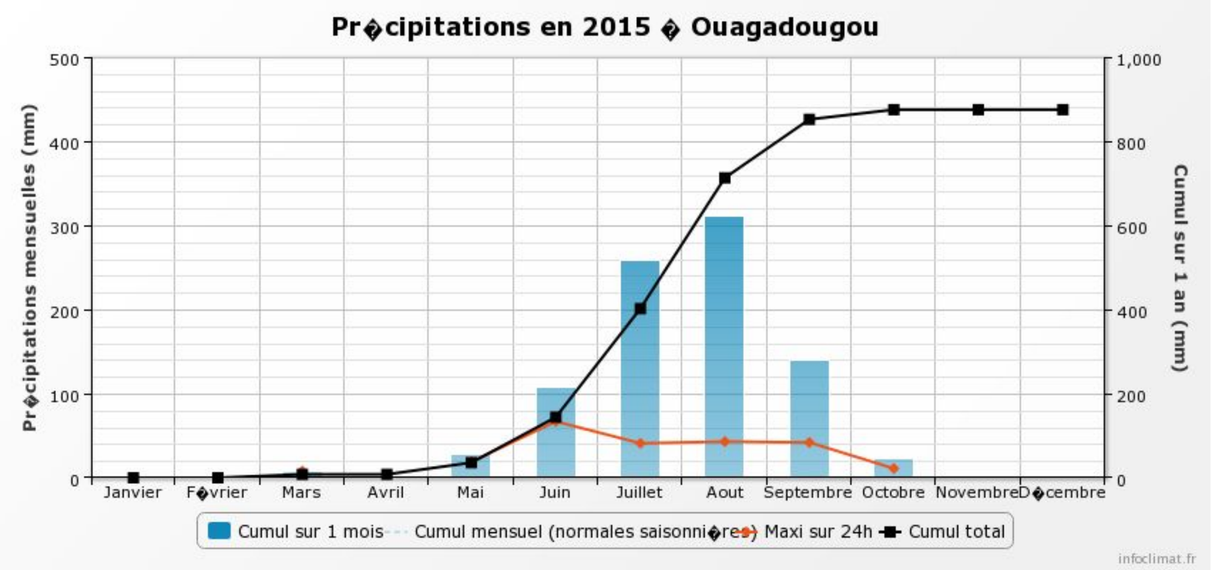

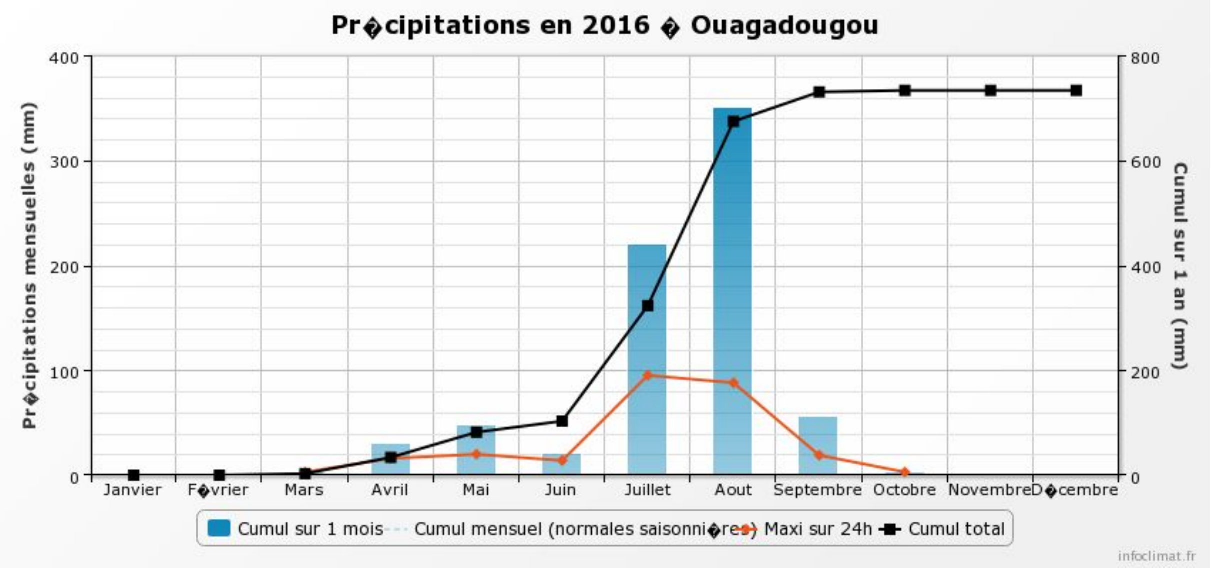

Supplement: Supplementary file 1 — Additional file 1. [file 40249_2020_675_MOESM1_ESM.pdf]
