# Supplementary material for: Impact of a community-based intervention on Aedes aegypti and its spatial distribution in Ouagadougou, Burkina Faso
Source: Infect Dis Poverty. 2020 Jun 5;9:61. doi: 10.1186/s40249-020-00675-6 (PMC7275586; doi:10.1186/s40249-020-00675-6)
Supplement: Supplementary file 2 — Additional file 2. Questionnaire Endline. [file 40249_2020_675_MOESM2_ESM.doc]

**Questionnaire Endline**

DVI_HCU_1 Numéro de l’étude

DVI_HCU_2 Code interviewer

DVI_HCU_3 quartier

DVI_HCU_4 secteur

DVI_HCU_5 Identifiant ménage (identique pour T0 T6 et T12)

DVI_HCU_6 "Avez-vous entendu parler de cette intervention par vos amis, voisins, des membres de la famille lors de causerie, porte à porte, activité communautaire, spectacle (théâtre) ?

DVI_HCU_7 Qu’avez-vous entendu au sujet de cette intervention?[champ libre]

DVI_HCU_8. remplissez le tableau suivant pour tous les membres de la famille

|  | | | | DVI_HCU_8_1  Numéro d'identification dans le ménage (XXXX ) | DVI_HCU_8_2  Age  (années/mois) | | DVI_HCU_8_3  genre | DVI_HCU_8_4  Statut dans la famille | DVI_HCU_8_5  Education | DVI_HCU_8_6  Occupation |
| --- | --- | --- | --- | --- | --- | --- | --- | --- | --- | --- |
| Répondant | | | |  |  |  |  |  |  |  |
| membre | | | |  |  |  |  |  |  |  |
| membre | | | |  |  |  |  |  |  |  |
| membre |  |  |  |  |  |  |  |  |  |  |
| membre |  |  |  |  |  |  |  |  |  |  |
| membre |  |  |  |  |  |  |  |  |  |  |
| membre |  |  |  |  |  |  |  |  |  |  |
| membre |  |  |  |  |  |  |  |  |  |  |
| membre |  |  |  |  |  |  |  |  |  |  |
| membre |  |  |  |  |  |  |  |  |  |  |
| membre |  |  |  |  |  |  |  |  |  |  |
| membre |  |  |  |  |  |  |  |  |  |  |
| membre |  |  |  |  |  |  |  |  |  |  |
| membre |  |  |  |  |  |  |  |  |  |  |
| membre |  |  |  |  |  |  |  |  |  |  |
| membre |  |  |  |  |  |  |  |  |  |  |
| membre |  |  |  |  |  |  |  |  |  |  |

| DVI_HCU_8_4 Statut dans la famille | | | | |
| --- | --- | --- | --- | --- |
| 1 | = | chef de famille | 7 | = conjoint/conjointe du chef de famille |
| 2 | = | Fils/ fille | 8 | = conjoint/conjointe du fils/fille du chef de famille |
| 3 | = | Petit-fils ou petite-fille du chef de famille | 9 | = conjoint/conjointe des petits-enfants du chef de famille |
| 4 | = | Frère/sœur du chef de famille | 10 | = belle-sœur/beau/frère du chef de famille |
| 5 | = | Mère/père du chef de famille | 11 | = belle-mère/beau-père du chef de famille |
| 6 | = | Membre de la famille étendue | (99) | = autre (spécifier)__________ |

| DVI_HCU_8_5  Education | DVI_HCU_8_6  Occupation | |
| --- | --- | --- |
| 1. pas scolarisé/pas alphabétisé    2.  pas scolarisé mais alphabétisé    3.  primaire (1 à 6 années de scolarité)    4.  secondaire-premier cycle (7 à 10 ans de scolarité)    5.  secondaire-deuxième cycle (11 à 13 ans de scolarité)    6.  diplôme professionnel/école des métiers    7.  Université (DEUG/licence)    8.  Université (Master1/Master2/doctorat/spécialisation médicale)    9.  éducation religieuse    10. éducation informelle    11. autre (précisez)    12. ne sait pas | 1. étudiant/écolier 2. retraité 3. ménagère 4. chômeur 5. employé du secteur privé 6. fonctionnaire 7. commerçant/propriétaire d’un commerce 8. travailleur non qualifié 9. travailleur qualifié 10. vendeur ambulant 11. fermier/berger 12. travailleur communautaire 13. petit commerce 14. pêcheur 15. secteur des services (chauffeur, service domestique, cuisinier, employé hôtel ou restaurant)   98. autre |  |

DVI_HCU_9 Est-ce que vous êtes capable de lire? Par exemple le journal

1. oui

2. oui, avec difficulté

3. non

DVI_HCU_10 Est-ce que le chef de ménage est capable de lire? Par exemple le journal (même s'il s'agit du chef de famille)

1. oui

2. oui, avec difficulté

3. non

DVI_HCU_11 Est-ce que la maitresse de maison est capable de lire? Par exemple le journal (même s'il s'agit du chef de famille)

1. oui

2. oui, avec difficulté

3. non

DVI_HCU_12 Quel type d’assurance santé possède la famille?

**CAP maladies fébriles, Paludisme, Dengue**

DVI_HCU_KAP1 Pouvez-vous citer des maladies qui peuvent donner de la fièvre?

1 Paludisme (QMFIE_1a)

2 Dengue (QMFIE_1b)

3 Méningite (QMFIE_1c)

4 Infection des voies

respiratoires (QMFIE_1d)

5 Pneumonies (QMFIE_1e)

6 Dysenterie (QMFIE_1f)

7 Typhoïde (QMFIE_1g)

8 Fièvre jaune (QMFIE_1h)

9 Ébola (QMFIE_1i)

10 Autre (QMFIE_1j)

10 Autre, précisez (QMFIE_1jAutre)

11 Ne sait pas (QMFIE_1k)

DVI_HCU_KAP2 Pouvez-vous nous dire si les maladies fébriles sont graves chez les personnes suivantes

Toutes les personnes (QMFEB_1a)

Enfants de moins de 5 ans (QMFEB_1b)

Femmes enceintes (QMFEB_1c)

Adultes (QMFEB_1d)

Personnes âgées (QMFEB_1e)

**1. Perceptions et attitudes envers la dengue**

DVI_HCU_KAP3 Avez-vous déjà entendu parler de la fièvre dengue (ou du palu-dengue)?

1. oui
2. non

99. ne sait pas

DVI_HCU_KAP4 selon vous, est-ce que la dengue est une autre forme de paludisme?

1. oui
2. non

99. ne sait pas

DVI_HCU_KAP5 Selon vous, est-ce que la dengue est une maladie grave?

1. oui
2. non

99. ne sait pas

DVI_HCU_KAP6. Quels sont les symptômes de la fièvre dengue?

a Fièvre (DVI_HCU_KAP6_2a)

b Rougeur au niveau du visage (DVI_HCU_KAP6_2b)

c Rash ou éruption cutanée (DVI_HCU_KAP6_2c)

d Douleur dans les yeux (DVI_HCU_KAP6_2d)

e Céphalées (DVI_HCU_KAP6_2e)

f Fatigue/faiblesse (DVI_HCU_KAP6_2f)

g Saignements (nez, bouche, autre,..) (DVI_HCU_KAP6_2g)

h Mal de gorge (DVI_HCU_KAP6_2h)

i Anorexie (DVI_HCU_KAP6_2i)

j Bouche sèche (DVI_HCU_KAP6_2j)

k Pieds/mains froides (DVI_HCU_KAP6_2k)

l Douleurs articulaires/ musculaires (DVI_HCU_KAP6_2l)

m Nausées ou Vomissements (DVI_HCU_KAP6_2m)

n Écoulement nasaux/symptômes d’infection respiratoire supérieure/Toux (DVI_HCU_KAP6_2n)

o Diarrhée (DVI_HCU_KAP6_2o)

p Autre (DVI_HCU_KAP6_2p)

q Ne sait pas/pas certain (DVI_HCU_KAP6_2q)

DVI_HCU_KAP2.3. Selon vous comment se transmet la dengue

a Par les moustiques (DVI_HCU_KAP6_3a)

b Par les moustiques différents de ceux du paludisme (Aèdes) (DVI_HCU_KAP6_3b)

c Par le contact avec les membres de la famille/communauté infectés

(DVI_HCU_KAP6_3c)

d Par des aliments non hygiéniques/de l’eau non bouillie (DVI_HCU_KAP6_3d)

e Par le mauvais temps (DVI_HCU_KAP6_3e)

f En visitant des régions agricoles ou on trouve des vaches et des porcs (DVI_HCU_KAP6_3f)

g En visitant des aires ou des gens déplacés vivent (DVI_HCU_KAP6_3g)

h Par la peau (DVI_HCU_KAP6_3h)

i Par les rapports intimes (DVI_HCU_KAP6_3i)

j Dans l’air (DVI_HCU_KAP6_3j)

k Par l’eau (DVI_HCU_KAP6_3k)

l Autre (DVI_HCU_KAP6_3l)

Spécifiez (DVI_HCU_KAP6_3BIS)

m Ne sait pas/incertain (DVI_HCU_KAP6_3m)

DVI_HCU_KAP7. Est-ce que quelqu’un dans votre ménage (incluant vous-même) a souffert de la fièvre dengue dans les 6 derniers mois?

1. Oui
2. Non (sauter à la question DVI_HCU_KAP8)

9. ne sait pas/incertain (sauter à la question DVI_HCU_KAP8)

DVI_HCU_KAP8_1 Qui a souffert de fièvre dengue dans le ménage et à quelle date? (réponses multiples permises)

| Liste | | DVI_HCU_8_1  Numéro d'identification dans le ménage (XXXX ) | DVI_HCU_KAP8_1  Le diagnostic confirmé par confirmé par un agent de santé ou un examen de laboratoire Oui/Non | DVI_HCU_KAP8_2  Nombre d’épisodes | Quand ? | | |  |  | | | |  |
| --- | --- | --- | --- | --- | --- | --- | --- | --- | --- | --- | --- | --- | --- |
|  |  |  |  |  | DVI_HCU_KAP8_3  Dernier épisode | DVI_HCU_KAP8_4  Épisode antérieur | DVI_HCU_KAP8_5  Ne sait pas | DVI_HCU_KAP8_6 | DVI_HCU_KAP8_7 | DVI_HCU_KAP8_8 | DVI_HCU_KAP8_9 | DVI_HCU_KAP8_10 | DVI_HCU_KAP8_11 à DVI_HCU_KAP8_13 |
| Répondant | |  |  |  |  |  |  |  |  |  |  |  |  |
| conjoint | |  |  |  |  |  |  |  |  |  |  |  |  |
| Autre | 1 |  |  |  |  |  |  |  |  |  |  |  |  |
| Autre | 2 |  |  |  |  |  |  |  |  |  |  |  |  |
| Autre | 3 |  |  |  |  |  |  |  |  |  |  |  |  |
| Autre | 4 |  |  |  |  |  |  |  |  |  |  |  |  |
| Autre | 5 |  |  |  |  |  |  |  |  |  |  |  |  |
| Autre | 6 |  |  |  |  |  |  |  |  |  |  |  |  |
| Autre | 7 |  |  |  |  |  |  |  |  |  |  |  |  |
| Autre | 8 |  |  |  |  |  |  |  |  |  |  |  |  |
| Autre | 9 |  |  |  |  |  |  |  |  |  |  |  |  |
| Autre | 10 |  |  |  |  |  |  |  |  |  |  |  |  |
| Autre | 11 |  |  |  |  |  |  |  |  |  |  |  |  |
| Autre | 12 |  |  |  |  |  |  |  |  |  |  |  |  |
| Autre | 13 |  |  |  |  |  |  |  |  |  |  |  |  |
| Autre | 14 |  |  |  |  |  |  |  |  |  |  |  |  |
| Autre | 15 |  |  |  |  |  |  |  |  |  |  |  |  |

DVI_HCU_KAP8_6 Où ont consulté les membres de votre famille y compris vous qui ont souffert de dengue?

DVI_HCU_KAP8_7. Si oui, quelle est la date de la plus récente infection de dengue?

DVI_HCU_KAP8_8. Si oui, avez-vous été hospitalisé pour la dengue?

DVI_HCU_KAP8_9. Quelle est la date de la plus récente hospitalisation pour la dengue?

DVI_HCU_KAP8_10. Quelle est la durée de la plus récente hospitalisation pour la dengue? (jours)

DVI_HCU_KAP8_11 Indiquez quels facteurs ont influencé votre décision de consulter . (plusieurs réponses permises)

DVI_HCU_KAP8_12 SVP indiquez la raison de ne pas être allé au CSPS  pour la fièvre dengue pour les individus qui ont rapporté avoir souffert de dengue dans les questions

DVI_HCU_KAP8_13. Si oui, pour vous ou le membre de la famille ayant eu la Dengue avez-vous participé à l’étude « surveillance de la dengue

1 non

2 oui, premier épisode

3 oui, deuxième épisode

4. oui, troisième épisode

99. NSP

DVI_HCU_KAP9. Vous ou un membre de votre famille avez-vous visité le CSPS de votre quartier pour de la fièvre dans les 6 derniers mois ?

DVI_HCU_KAP10. Est-ce que quelqu’un est décédé de dengue dans votre ménage?

DVI_HCU_KAP11. Combien de personnes sont décédées de dengue dans chaque catégorie d’âge suivante

a Moins de 1 an : (nombre) (DVI_HCU_KAP11a)

b Enfant 1-5 ans: (nombre) (DVI_HCU_KAP11b)

c Enfant 6-10 ans: (nombre) (DVI_HCU_KAP11c)

d Enfant 11-18 ans: (nombre) (DVI_HCU_KAP11d)

e Adulte 19-54 ans: (nombre) (DVI_HCU_KAP11e)

f Adulte Plus de 54: (nombre) (DVI_HCU_KAP11f)

g Ne sais pas/incertain: (nombre) (DVI_HCU_KAP11g)

DVI_HCU_KAP12. Connaissez-vous personnellement quelqu’un qui a souffert de dengue à l’extérieur de votre ménage?

DVI_HCU_KAP.13. Connaissez-vous personnellement quelqu’un qui est décédé de dengue à l’extérieur de votre ménage?

DVI_HCU_KAP.14. Quelle est la fréquence de la fièvre dengue dans votre quartier?

DVI_HCU_KAP.15. Quelle est la gravité de la fièvre dengue chez les enfants de moins de 18 ans?

DVI_HCU_KAP.16 Quelle est la gravité de la fièvre dengue chez les adultes?

DVI_HCU_KAP.17. Quelles sont les probabilités que vous vous infectiez avec la dengue dans les 5 prochaines années?

DVI_HCU_KAP.18. Quelles sont les probabilités que les jeunes enfants de votre ménage (moins de 18 ans) s’infectent avec la dengue dans les 5 prochaines années?

**2. Perceptions et attitudes envers le Palu**

DVI_HCU_KAP.19 Selon vous comment se transmet le paludisme d’une personne à une autre?

1 Par la peau (QMPAL_1a)

2 Par les rapports intimes (QMPAL_1b)

3 Par les moustiques (QMPAL_1c)

4 Dans l’air (QMPAL_1d)

5 Par l’eau (QMPAL_1e)

6 Par la nourriture (QMPAL_1f)

7 Par les pluies (QMPAL_1g)

8 Consommation excessive d’huile, fruits sucrés ou de lait (QMPAL_1h)

9 Manque de sommeil/fatigue (QMPAL_1i)

10 Exposition au soleil (QMPAL_1j)

11 Ne sait pas (QMPAL_1k)

DVI_HCU_KAP.20 Pouvez-vous me citer les symptômes de paludisme?

1 Fièvre (QMSYM_1a)

2 Manque d’appétit (QMSYM_1b)

3 Vomissements (QMSYM_1c)

4 Frissons (QMSYM_1d)

5 Crises convulsives (QMSYM_1e)

6 Évanouissement (QMSYM_1f)

7 Maux de tête/céphalées (QMSYM_1g)

8 Courbatures (QMSYM_1h)

9 Diarrhée (QMSYM_1i)

10 Fatigue (QMSYM_1j)

11 Nausée (QMSYM_1k)

12 Pâleur (QMSYM_1l)

13 Douleur abdominale (QMSYM_1m)

14 Jaunisse (QMSYM_1n)

15 Ne sait pas (QMSYM_1o)

16 Autre (QMSYM_1p)

DVI_HCU_KAP.21 Selon vous, quelle est la cause du paludisme ?

Moustiques (QMCAU_1a)

Chaleur, soleil (QMCAU_1b)

Pluies (QMCAU_1c)

Alimentation (QMCAU_1d)

Manque d’hygiène (QMCAU_1e)

Eaux sales (QMCAU_1f)

Sorcellerie/mauvais esprits (QMCAU_1g)

Autre (QMCAU_1h)

DVI_HCU_KAP.22 Selon vous, est-ce que le paludisme est une maladie grave?

**3. Connaissances et pratiques sur les maladies febriles**

DVI_HCU_KAP.23 Si un adulte ou un enfant fait de la fièvre, que croyez-vous qu’il convienne de faire?

1 Attendre que les symptômes passent/Rien (Q2_FAI1)

2 Continuer à s’hydrater (Q2_FAI2)

3 Prendre des médicaments, si oui (Q2_FAI3)

lesquel? (Q2_FAI3Aut)

4 Consulter un centre de santé (Q2_FAI4)

5 Consulter tradipraticien (Q2_FAI5)

6 Consulter pharmacien/vendeur Rx (Q2_FAI6)

7 Prier/consulter un prêtre/pasteur/imam (Q2_FAI7)

8 Le couvrir (Q2_FAI8)

9 Autre (Q2_FAI9)

Spécifiez (Q2_FAI9Aut)

DVI_HCU_KAP.24 Si un adulte ou un enfant présente de la fièvre, qu’est ce qui vous inciterait à consulter?

Fièvre qui dure (Q2_CONS1)

Pâleur (Q2_CONS2)

Inconscience/évanouissement (Q2_CONS3)

Fatigue extrême (Q2_CONS4)

Déshydratation (Q2_CONS5)

Difficulté à respirer (Q2_CONS6)

Incapacité de manger ou boire (Q2_CONS7)

Personne qui n’urine plus (Q2_CONS8)

Vomissements/diarrhées qui ne cessent pas (Q2_CONS9)

Saignement (Q2_CONS10)

Jaunisse (Q2_CONS11)

Convulsions (Q2_CONS12)

Inquiétude des proches (Q2_CONS13)

Autre (Q2_CONS14)

précisez (Q2_CONS14Aut)

DVI_HCU_KAP.25 Si un adulte ou un enfant présente de la fièvre, qu’est ce qui vous empêcherait de consulter?

Capacités de payer (Q2_EMP1)

Distance avec le centre de santé (Q2_EMP2)

Qualité des soins dans le centre de santé (Q2_EMP3)

Difficultés pour se déplacer/pas de transport (Q2_EMP4)

Opposition de certains membres de la famille (Q2_EMP5)

Autre (Q2_EMP6)

Précisez(Q2_EMP6Aut)

DVI_HCU_KAP.26 Selon vous, quand vous allez consulter à cause de la fièvre, que croyez-vous que le soignant doit faire?

Prescrire des antipaludiques/ACT (Q2_SOI1)

Prescrire des antibiotiques (Q2_SOI2)

Donner des conseils (Q2_SOI3)

Faire des tests (Q2_SOI4)

Autre (Q2_SOI5)

Précisez (Q2_SOI5Aut)

Ne sait pas/incertain (Q2_SOI6)

DVI_HCU_KAP.27 Connaissez-vous des moyens de vous protéger et de protéger vos proches contre les maladies fébriles transmises par les vecteurs?

1 Dormir sous une moustiquaire (Q2_MOY1)

2 Recours aux postes de santé (Q2_MOY2)

3 Boire de l’eau propre (Q2_MOY3)

4 Se vacciner (Q2_MOY4)

5 Éviter le contact avec les gens malades (Q2_MOY5)

6 Éviter le contact avec les animaux (Q2_MOY6)

7 Se déparasiter (Q2_MOY7)

8 Prendre des vitamines (Q2_MOY8)

9 Prendre des médicaments préventifs (Q2_MOY9)

10 Utiliser des insecticides/répulsifs chimiques/spirales/raquettes (Q2_MOY10)

11 Aucune (Q2_MOY11)

DVI_HCU_KAP.28 Est-ce que vous connaissez des moyens de prendre soin de votre maison ou de votre cour pour éviter les maladies fébriles transmises par des vecteurs?

1 Pulvérisation intra-domicilaire (Q2_SOY1)

2 Utilisation de rideaux imprégnés (Q2_SOY2)

3 Grillage antimoustique (Q2_SOY3)

4 Utiliser des insecticides/répulsifs chimiques/spirales(Q2_SOY4)

5 Utiliser de la poudre (épandage) (Q2_SOY5)

6 Nettoyer la concession (Q2_SOY6)

7 Recouvrir les contenants d’eau (Q2_SOY7)

8 Se défaire des objets qui peuvent accumuler de l’eau ou les vider (Q2_SOY8)

9 Changer l’eau des pots à fleurs (Q2_SOY9)

10 Aucune (Q2_SOY10)

DVI_HCU_KAP.29 Est-ce que quelqu’un dans ce ménage fait quelque chose ou dépensé de l’argent pour éviter spécifiquement d’être infecté par les maladies transmises par les vecteurs

DVI_HCU_KAP.30. Est-ce que vous ou votre famille prenez les précautions suivantes pour éviter les maladies transmises par les vecteurs?

a Recouvrir les contenants d’eau (DVI_HCU_KAP.30a)

b Se défaire des objets qui peuvent accumuler de l’eau ou les vider (DVI_HCU_KAP.30b)

c Raquette électrique (DVI_HCU_KAP.30c)

d Utilisation d’insecticides/répulsifs chimiques/spirales (DVI_HCU_KAP.30d)

e Éviter les aires avec la dengue/paludisme (DVI_HCU_KAP.30e)

f Utilisation de MILDA (DVI_HCU_KAP.30f)

g Utilisation de grillage antimoustique aux fenêtres (DVI_HCU_KAP.30g)

h Changer l’eau dans les pots à fleurs (DVI_HCU_KAP.30h)

i Autre (DVI_HCU_KAP.30i)

Spécifiez (DVI_HCU_KAP.30BIS)

j Ne sait pas/incertain (DVI_HCU_KAP.30j)

k Pulvérisation intra-domicilaire (DVI_HCU_KAP.30k)

l Utilisation de rideaux imprégnés (DVI_HCU_KAP.30l)

m Utiliser de la poudre (épandage) (DVI_HCU_KAP.30m)

n Nettoyer la concession (DVI_HCU_KAP.30n)

o Changer l’eau des pots à fleurs (DVI_HCU_KAP.30o)

**Pratiques de préventions adoptées par le ménage**

DVI_HCU_KAP.31. À quelle fréquence les membres du ménage utilisent des insecticides chimiques?

DVI_HCU_KAP.31BIS. si autre, précisez

DVI_HCU_KAP.32. Pendant combien de mois/semaines par année prenez-vous des précautions pour éviter le moustique qui transmet la dengue ?

DVI_HCU_KAP.32Sem Semaine(s)

DVI_HCU_KAP.32Mois Mois

DVI_HCU_KAP.33. Combien d’heures sont consacrées par semaine dans ce ménage aux activités de contrôle du vecteur de la dengue?

DVI_HCU_KAP.34. Si vous conservez de l’eau dans des contenants dans votre maison ou dans votre cours, est-ce que vous les couvrez?

DVI_HCU_KAP.35. dans votre maison ou dans votre cour y a-t-il des récipients/déchets qui accumulent de l’eau de pluie

DVI_HCU_KAP.36. Combien d’argent votre ménage dépense-t-il par an pour le contrôle des vecteurs (ex : insecticide, répulsif, contenants spéciaux,…)

DVI_HCU_KAP.37. Si les membres du ménage ne font pas d’activités de contrôle de vecteurs, pourquoi?

DVI_HCU_KAP.37BIS. si autre, précisez

DVI_HCU_KAP.38 Est-ce qu'à n'importe quel moment au cours des 12 derniers mois, quelqu'un est venu dans votre logement pour pulvériser les murs intérieurs contre les moustiques ?

DVI_HCU_KAP.39 Est-ce que votre ménage a des moustiquaires qui peuvent être utilisées pour dormir ?

DVI_HCU_KAP.40_MOUSNbr Combien de moustiquaires possédez-vous dans votre ménage ?

DVI_HCU_KAP.40_MOUSImpr Est-ce une moustiquaire imprégnée?

DVI_HCU_KAP.40_MOUSPers_ Combien de personnes ont dormi sous cette moustiquaire la nuit dernière?

DVI_HCU_KAP.40_MOUSPers Nombre

DVI_HCU_KAP.40_PersMais combien de personnes ont dormi dans cette maison la nuit dernière?

DVI_HCU_KAP.40_PersMaisNbr Nombre

Pour vous, quelle est l’utilité d’une moustiquaire?

1 Tuer les moustiques (Q2_MOU1)

2 protéger des piqures d’insectes (Q2_MOU2)

3 protéger du paludisme (Q2_MOU3)

4 mieux dormir (Q2_MOU4)

5 servir de rideaux ou de draps de lits (Q2_MOU5)

6 servir de filets de pêche (Q2_MOU6)

7 aucune (Q2_MOU7)
